# Novel characterisation of microplastics and other contaminant particles using new scanning electron microscopy technologies

**DOI:** 10.1038/s41598-025-27992-w

**Published:** 2025-11-29

**Authors:** Max Webb, Jim Buckman, Bérénice Ratouit, Jennifer Scott, Juliane Bischoff, Thao Thanh Le, Tony Gutierrez, Thomas Wagner, Huong Thi Thuy Ngo, Michel Kaiser, Ryan Pereira

**Affiliations:** 1 The Lyell Centre, School of Energy, Geoscience, Infrastructure and Society, Heriot-Watt University, Edinburgh, UK; 2 Institute of GeoEnergy Engineering, School of Energy, Geoscience, Infrastructure and Society, Heriot-Watt University, Edinburgh, UK; 3 Department of Health Engineering, University of Poitiers, Poitiers, France; 4 Institute of Mechanical, Process and Energy Engineering, School of Engineering and Physical Science, Heriot-Watt University, Edinburgh, UK; 5 Environmental Chemistry and Ecotoxicology Lab, Phenikaa University, Yen Nghia, Ha Dong, Hanoi, 12116 Vietnam; 6 Faculty of Biotechnology, Chemistry and Environmental Engineering, Phenikaa University, Yen Nghia, Ha Dong, Hanoi, 12116 Vietnam

**Keywords:** Microplastics, SEM-BEX, Environmental contamination, Environmental screening, Vietnam, Environmental sciences, Environmental impact, Scanning electron microscopy

## Abstract

Scanning electron microscopy with combined backscattered electron and X-ray imaging (SEM-BEX) represents a new way to conduct direct visual assessment and elemental characterisation of particles in environmental samples up to 18× faster than standard scanning electron microscopy techniques. SEM-BEX provides all element maps combined with back-scattered electron microscopy, which allows the detection of the elemental composition of individual particles, such as microplastics and others, in a semi-automated fashion. Detection of characteristic elements leads to further quantification of specific particles. This provides improved versatility compared to the elemental scans provided by standalone energy-dispersive X-ray spectroscopy (EDX) techniques and strongly increases multi-sample throughput speeds. The applications of this new technology for environmental contamination research include estimations on the morphology and distribution of microplastics and other particles alongside their interaction with micro-organisms and toxicity assessments by tracing the transport of trace metals through the environment on both contaminant (microplastics) and natural (suspended sediment) vectors. This study used filtered samples from the Cát Bà Islands of Viet Nam to assess the applicability of SEM-BEX to environmental contamination research, our results indicate that microplastics and other particles can be physically and chemically characterised across all samples down to a minimum particle size of 5 µm^2^, in addition, we show that SEM-BEX is particularly powerful for identifying transparent fragments that are otherwise missed by optical studies. Trace metals were also detected, including Cr, Ti, and Hg, which might be due to pigment composition in paints or plastics, or adsorbed onto particles from the environment. Ultimately, SEM-BEX has broad applications as a rapid screening tool for environmental assessments in identifying contamination hot-spots before conducting particle-specific analysis (such as Raman spectroscopy). Further potential also exists to accelerate screening using artificial intelligence machine learning.

## Introduction

The characterisation of microplastic morphology and distribution has become an important issue, largely due to their abundance (6.3 billion metric tons globally)^1^, small size (less than 5 mm) making them easily ingestible^2^, and potential toxicity to the environment and biota^3^. Microplastics are generally classified into five key morphologies (fragments, spheres or pellets, films, foams, and fibres)^4^ and a range of polymer-types, such as polyamide (nylon), high-density and low-density polyethylene (HDPE, LDPE), polyethylene terephthalate or polyester (PET), polyvinyl chloride (PVC), polystyrene (PS), polypropylene (PP), and rubber. On top of microplastic pollution the accumulation of other environmental contaminant particles (e.g., paint flecks) and the fixation of biofilms, toxins, and trace metals to these particles potentially introduces additional environmental and pathogenic hazards^5^. Furthermore, the breakdown of microplastic particles (1 µm–5 mm) into smaller nanoplastic particles (< 1 µm) through thermal, mechanical, and biological degradation^6^ also means that determining the size, shape, and abundance of plastic particles within a given environmental sample often requires the use of scanning electron microscopy (SEM) capable of imaging submicron particles^7^.

For the detection of microplastics, optical light microscopy is often used as an initial screening method prior to applying SEM techniques for further analysis. SEM can then provide additional information on morphology, typology, and origin of the plastic particles^8^, with SEM systems traditionally supporting three different techniques for plastic analysis: (1) Secondary electron (SE) imaging to distinguish the physical and morphological nature of plastics, (2) Backscattered electron (BSE) imaging to determine the topography and, to an extent, the relative chemical composition of the plastic (with samples containing elements of a higher atomic number providing more backscattered electrons and a brighter image, e.g., PVC), and (3) Electron dispersive X-ray (EDX) spectrometry for elemental analysis, providing compositional data and the differentiation of plastics from organic matter and inorganic minerals^8–10^. In addition, EDX combined with SEM imaging can be used to provide further indirect information, such as nitrogen peaks on PP, PE, and PS particles, acting as a proxy for biomass and bioaccumulation^11^, or the detection of titanium dioxide nanoparticles (TiO_2_-NPs) on the surface of plastics^12^. Despite the wide range of successful applications of SEM–EDX to microplastic studies, slow scanning rates mean that these studies often involve individual microplastic particle analysis with additional time spent manually moving analytical sites between particles. In addition, the separation of individual microplastics further increases analytical time and their extraction from the natural sample load of a given environment (e.g., soils, filter papers, etc.) removes context clues on the overall pollution potential of microplastics (e.g., the abundance of toxic trace metals adsorbed to the microplastics compared with environmental background levels or their interaction with biota).

To address this technological challenge, we have developed a semi-automated approach to characterising microplastics and other particles using a state-of-the-art Unity-BEX detector coupled to an SEM (SEM-BEX) with additional cross-referencing using optical microscopy. SEM-BEX is a new SEM imaging technique using the Oxford Instruments Unity detector (https://nano.oxinst.com/unity). We use this technology advancement for improved and rapid detection of microplastics and further quantification of trace metals. The Unity detector contains both backscattered electron and X-ray imaging (BEX) sensors (silicon drift detectors—SDDs) within a single detector, meaning that elemental, morphological, and topographic data can all be rapidly collated. Compared to traditional SEM–EDX methods that take hours, the SEM-BEX method defined here analyses samples in approximately 20 min and presents them in high-resolution false-colour maps. Individual elements can be identified, quantified (when paired with an attached X-Max^N^ 150 mm Energy Dispersive X-ray detector), assigned false-colours for improved visualisation, and layered together as all element maps with integrated BSE imagery. This concurrent acquisition and layering of BSE images and elemental maps results in a new dataset unique to SEM-BEX. In addition, the combination of multiple sensors within one detector reduces the time spent switching between and optimising multiple detectors (e.g., SE, BSE, EDX) and removes shadowing effects from sample topography that is a common issue in standalone EDX detectors. The treatment of samples presented here is also novel as the fast, high-resolution scanning rates from the Unity detector allows us to analyse entire environmental samples (including suspended sediment, organic matter, microplastics, and other contaminant particles) in a fraction of the time taken to analyse individual particles during traditional SEM–EDX methods (~ 20 min per sample). This also eliminates the need for separation of microplastics and other particles from the surrounding matrix, a process that can be time-consuming and is potentially destructive. The BEX system has been successfully used for the characterisation and quantification of wear debris from periprosthetic tissue^13^ and heavy minerals during sedimentary provenance analysis^14^ but has not previously been applied to microplastics and environmental contamination research in natural samples, making this a unique opportunity to introduce a new way to rapidly assess and screen environmental samples.

Other SEM methodologies have been proposed for automated mapping of microplastics, such as QEMSCAN (Quantification and Evaluation of Minerals by Scanning Electron Microscopy)^15^. The QEMSCAN methodology also uses combined BSE and EDX and was initially designed to conduct automated mineralogy mapping of geological samples. This technique was used to test automated mapping of plastics, sand grains, and organic matter using samples containing predefined percentages of each^15^. Results from that study showed a significant over-quantification of plastic particles (up to 30% more plastic detected than was known to be present in the fabricated samples), likely due to the mis-identification of organic matter, indicating that over-automation or full reliance on automation should be treated with caution.

To test the potential of the new Unity detector and SEM-BEX for integrated environmental studies and to build upon previous attempts to automate plastic identification and mapping (e.g., QEMSCAN), this study utilised a set of eight environmental filter paper samples taken from the Cát Bà islands of northern Viet Nam. The samples underwent no further separation of particles (e.g., microplastics) from other environmental material (e.g., sediment, organic matter, etc.), thereby allowing us to test the efficacy of the SEM-BEX system for direct visualisation and characterisation of complex environmental matrices. This method also aims to optimise the analytical process and minimize sample preparation time.

Here we provide a novel workflow for the rapid visualisation and assessment of particles (including microplastics) within environmental samples using SEM-BEX alongside semi-automated quantification.

## Results

**Fig. 1:**
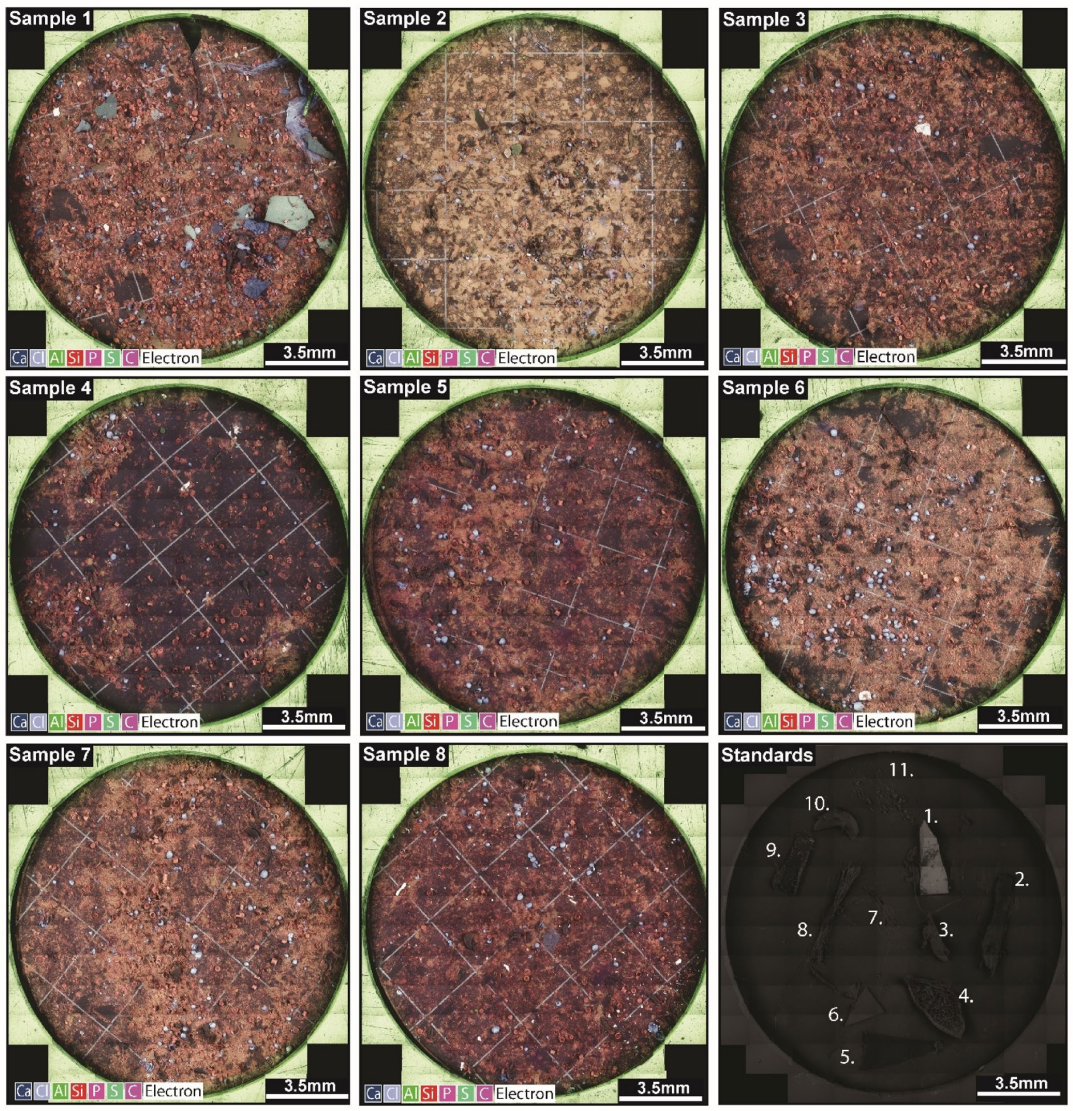
Imaging microplastics and other contaminant particles using SEM-BEX. All element maps for membrane filters containing environmental material collected from Cát Bà, Viet Nam and analysed using SEM-BEX. The SEM-BEX records all elemental data during scanning and key elements have been highlighted here (calcium [Ca], chlorine [Cl], aluminium [Al], silica [Si], phosphorous [P], sulfur [S], carbon [C]), electron refers to the combined BSE aspect of these images and represents material that doesn’t correspond to the chosen elements. Numbers on the standards slide refers to examples of virgin and other known plastics used to test the microplastic speciation capabilities of SEM-BEX: (1) clear PVC fragment, (2) brown HDPE fragment, (3) clear epoxy resin fragment, (4) white PS ball, (5) clear PE fragment, (6) clear PVA fragment, (7) white polyester fibres, (8) white polyester-cotton blend fibres, (9) blue PP fibres, (10) clear PU granule, white PET powder.

**Fig. 2:**
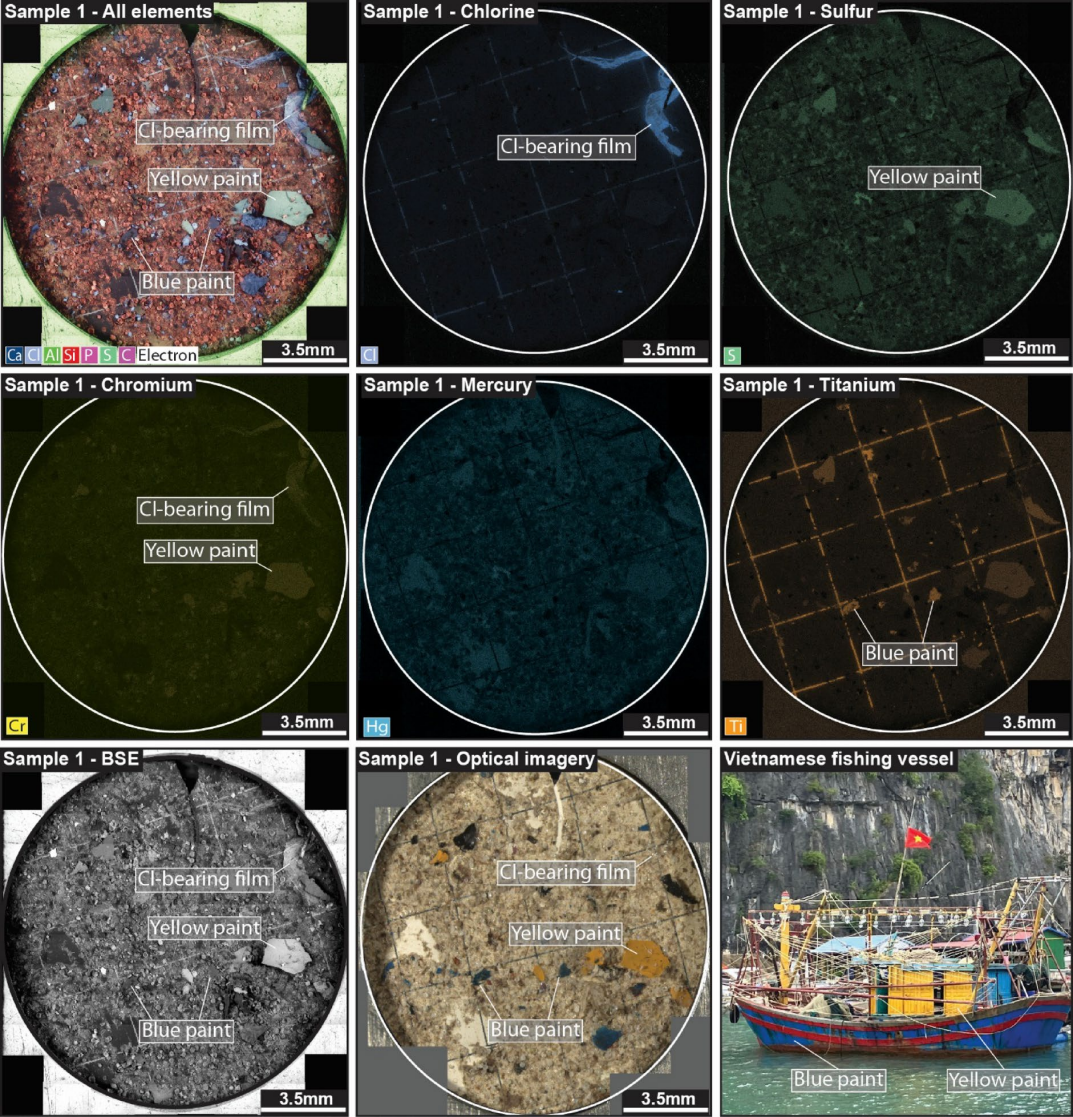
Characterising particles using SEM-BEX. All elements map for Sample 1 along with Cl, S, Cr, Hg, and Ti maps showing the identification of Cl-bearing plastic films (chlorine map) along with the presence and relative concentration of trace metals within both the film and paint flecks (including trace metals such as Hg and Cr). Also shown is an optical microscopy image of the sample to further aid distinction between particles (e.g., microplastics and paint flecks), such as those commonly used on Vietnamese fishing vessels in Cát Bà (bottom right image).

Initial analysis of standard materials using known polymer-types (outlined in Methods; Fig. 1) indicates that the SEM-BEX fast scanning function is not suitable to differentiate between all polymer types. Our results show that PVC and other chlorinated plastics are readily identifiable using chlorine maps (Table 1; Fig. 2), in addition PET may be speciated on a case-by-case basis when utilising a standalone EDX detector (PET contains a higher wt% of Oxygen than other plastics). However, this needs to be used in combination with a visual assessment of the plastic particle as increased Oxygen can also be present in weathered examples of other polymer-types (e.g., HDPE, PS, PP, PU, PE) that are otherwise indistinguishable from each other using this technique. Despite this, an important strength of this study is that whilst limitations exist in identifying distinct polymer types for microplastics using the SEM-BEX fast-scanning method, it has been effective in distinguishing contaminant particles (which here includes non-speciated microplastics, paint flecks, rubber, and rust fragments) from sediment and organic matter by characterisation of their size, shape, and chemistry, along with their true colour (using optical microscopy). In addition, there are many instances in which these contaminant particles can be distinguished from each other both qualitatively (e.g., blue and yellow paint flecks vs. clear plastic films; Fig. 2) and quantitatively (e.g., Fe-bearing rust fragments vs. S-bearing vulcanised black rubber fragments).

**Table 1:** Counts for Cl-bearing microplastics and other contaminant particles from all samples using the automated Fiji (ImageJ) approach including average size (with 95% CI), % area coverage for the entire sample, and average circularity index (with 95% CI). Also shown are particle counts using manual identification based on BSE and optical images for comparison of automation effectiveness.

Feature	Automated characterisation			
	Count	Average size (mm^2^)	%Area	Average circularity
Sample 1 (C.CB1.S.SEM1)				
Cl-bearing microplastic	3	1.108 ± 0.997	1.164	0.123 ± 0.109
Paint/rubber	13	0.299 ± 0.330	1.360	0.028 ± 0.012
Other particles	2	0.068 ± 0.030	0.048	0.320 ± 0.270
Total	18	Manual characterisation total	69	
Sample 2 (CB3.1.SEM1)				
Other particles	16	0.044 ± 0.024	0.244	0.094 ± 0.036
Total	16	Manual characterisation total	15	
Sample 3 (CB3.2.B.SEM2)				
Cl-bearing microplastic	1	0.057 ± 0.000	0.020	0.106 ± 0.000
Other particles	17	0.031 ± 0.031	0.184	0.426 ± 0.072
Total	18	Manual characterisation total	17	
Sample 4 (CB3.2.S.SEM1)				
Other particles	27	0.014 ± 0.004	0.132	0.474 ± 0.059
Total	27	Manual characterisation total	14	
Sample 5 (CB3.3.B.SEM1)				
Cl-bearing microplastic	1	0.014 ± 0.000	0.005	0.205 ± 0.000
Total	1	Manual characterisation total	16	
Sample 6 (CB3.3.B.SEM2)				
Other particles	4	0.034 ± 0.027	0.048	0.339 ± 0.270
Total	4	Manual characterisation total	5	
Sample 7 (CB3.3.B.SEM3)				
Cl-bearing microplastic	30	0.005 ± 0.002	0.055	0.236 ± 0.043
Other particles	18	0.003 ± 0.000	0.020	0.180 ± 0.031
Total	48	Manual characterisation total	16	
Sample 8 (CB3.3.M.SEM1)				
Cl-bearing microplastic	1	0.065 ± 0.000	0.023	0.109 ± 0.000
Other particles	101	0.005 ± 0.001	0.17	0.454 ± 0.031
Total	102	Manual characterisation total	107	

Bulk sample SEM-BEX imaging confirms that all eight filter paper samples analysed here contained contaminant particles alongside widespread environmental material (Fig. 1). The occurrence of these contaminant particles ranges from a single fragment in the least contaminated sample (Sample 5) to 102 individual particles, including non-speciated microplastic fragments and films, paint flecks, vulcanised rubber fragments, and metal filings (e.g., rust) in the most contaminated (Sample 8; Table 1). These particles were visually characterised using Fiji (ImageJ)^16^ and range in size from 2.3 mm^2^ to 5 µm^2^ with circularity indices ranging from 0.8 to 0.01 (where 1 = a perfect circle and 0 = a straight line; Table 1). Chlorine elemental maps were used to detect chlorinated plastics (e.g., PVC) in four out of eight samples (Table 1), which occurred in the shape of fragments, fibres, and colourless Cl-bearing films, such as the kind used throughout Viet Nam in PVC plastic bags. These colourless films represent the largest microplastic fragments observed in this study (~ 2.3 mm^2^; Sample 1; Table 1), despite this, their colourless nature makes them difficult to image under standard optical microscopy without the assistance of SEM-BEX. Any additional microplastic types along with other non-organic particles were classified as ‘other contaminants’ due to the inability of SEM-BEX to distinguish individual polymer types. These ‘other contaminants’ were distinguished from each other based on their shape, surface texture, and secondary confirmation using optical imagery. Blue and yellow paint flecks were observed in two of the samples (Table 1) using optical microscopy and distinguished from surrounding material using SEM-BEX calcium (Ca) maps (with Ca hotspots associated with the blue paint flecks in these samples) and a combination of sulfur (S), chromium (Cr), and lead (Pb) maps (which are here associated with yellow paint flecks—Fig. 2). In addition, vulcanised black rubber fragments were observed in one sample collected from a harbour area (Sample 1; Table 1) based on enrichment in the S map and further distinguished from yellow paint flecks based on its morphology and colour under optical microscopy.

**Fig. 3:**
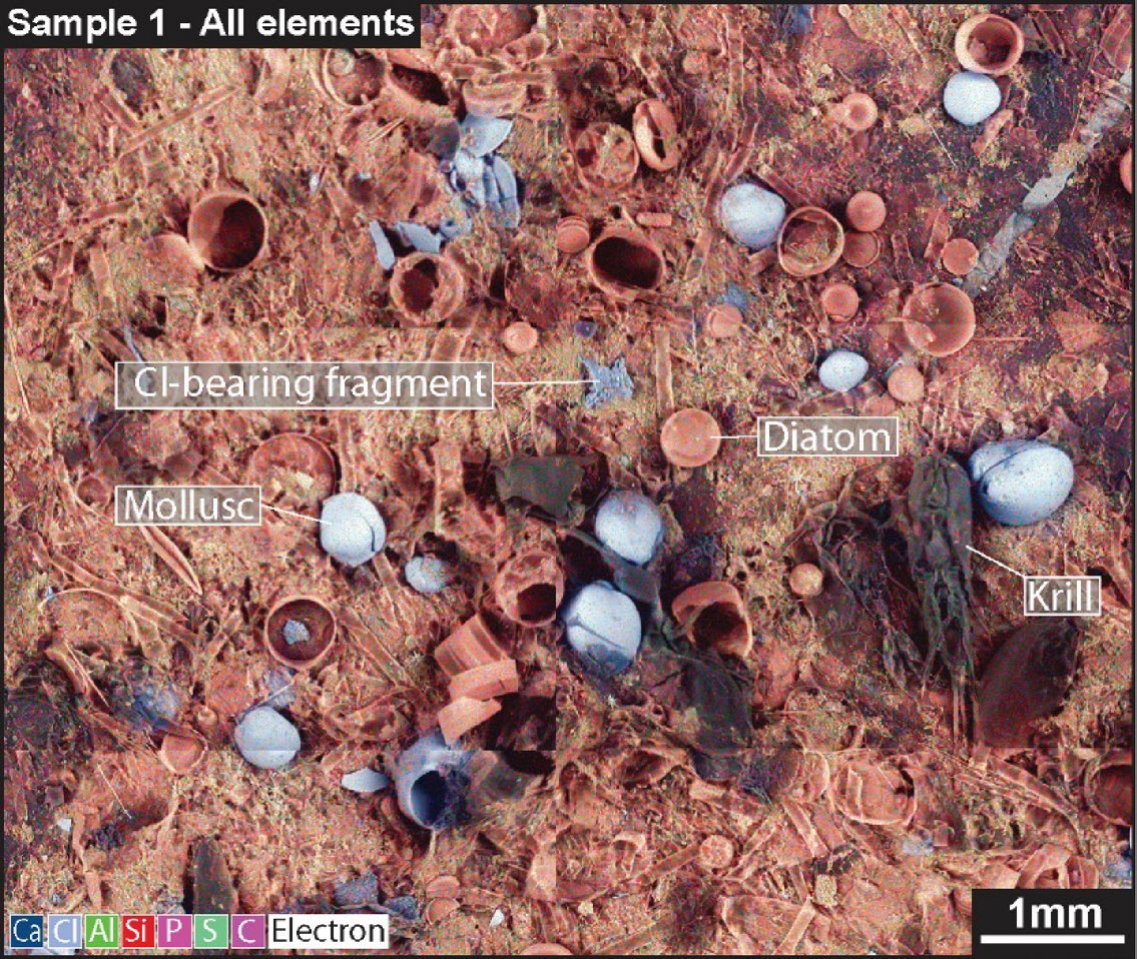
Contaminant particles in the environment. Zoomed-in all element map for Sample 1 showing the comparable size and shape amongst contaminant particles (Cl-bearing microplastic fragment shown here), diatoms, and molluscs from within the sampling environment. Also shown is the carapace of a larger zooplankton (krill).

To test the efficacy of automated characterisation of contaminant particles using Fiji (ImageJ) and SEM-BEX imagery, a parallel study was performed on the same samples using manual optical microscope and standard BSE imagery. This approach used Fiji (ImageJ) to manually draw around observed contaminant particles and extract the same physical characteristics (size, shape, abundance) but did not utilise the ‘analyse particles’ function to automatically characterise particle abundance and morphology. The results show that for half of the samples (samples 2, 3, 6, and 8; Table 1) the automated Fiji (ImageJ) approach successfully selected comparable numbers of particles as the manual approach (± 10%). In the remaining samples, however, the automated approach either under-quantified (e.g., Sample 5: automated approach n = 1; manual approach n = 16) or over-quantified (e.g., Sample 7: automated approach n = 48; manual approach n = 16) the number of contaminant particles present in a sample (Table 1). This is ultimately due to the ‘in-situ’ nature of the particles within other environmental material (e.g., sediment, organic matter, shells) meaning that Fiji (ImageJ) mis-characterises environmental material (e.g., shells) as contaminant particles (resulting in over-quantification) or ignores contaminants that share shape, size, and elemental characteristics with the environmental matrix (resulting in under-quantification). To mitigate for this uncertainty, we include an optical microscopy step within the proposed workflow (Fig. 3), whereby these standard light images can be used to further distinguish particles selected by Fiji (ImageJ) as either contaminants (e.g., colourful microplastics) or environmental (e.g., sediment).

The capability of SEM-BEX to scan all elements present in a sample allows for the identification and quantification of key elements of interest, including trace metals. In the samples analysed, all eight contained ~ 0.5–2 wt% titanium (Ti) and iron (Fe), respectively, based on coeval analysis using the attached X-Max^N^ 150 mm EDX detector), seven contained molybdenum ([Mo] ~ 1–2 wt%), five contained mercury ([Hg] ~ 1–2 wt%), and three contained trace amounts of tantalum (Ta) and magnesium (Mg). Using the X-Max^N^ 150 mm EDX detector we determined that whilst certain trace metals were also present in the plastic standards (which include coloured and uncoloured virgin plastics outlined in the “Methods” section), these were all in quantities of at least one order of magnitude lower than the environment samples (< 0.1 wt%; Fig. 1; Table 2). To understand the trace metal loads across individual particles, a focused analysis of Sample 1 was conducted, which was found to contain the largest contaminant particles (Table 2). Elemental maps for this sample showed the presence of chromium (Cr) and lead (Pb) within a Cl-bearing film and throughout the yellow paint flecks, Hg distributed throughout the sample, and Ti within the paint flecks and other contaminant particles (Ti also appears to be a component of the grid lines on the membrane filters; Fig. 2). Sources of these elements may be associated the manufacture of the microplastics and paints (with Cr and TiO_2_ commonly used as colourants) or with local shipping/boat traffic (e.g., diesel spillage, wearing of brakes, tyres, and batteries). It should be noted that at the accelerating voltage used in this study (20 kV) sample penetration depths of ~ 1–2 µm are expected during the SEM-BEX scan (depending on the sample material), meaning that any trace metals identified likely represent those adsorbed into the particles themselves and not just present on the surface or those that were present during its manufacture. This penetration depth also means that any biofilms present on a particles surface may interact with its elemental spectra, however, no obvious biofilms were observed during optical analysis of these samples.

**Table 2:** X-MaxN 150 mm energy dispersive X-ray (EDX) analytical results showing wt% of elements present in Sample 1 (Fig. 2) and the plastic standards sample (Fig. 1).

Sample 1			Sample 1			Sample 1			Plastic standards		
Whole sample			Cr-bearing paint fleck			Ti-bearing microplastic			Whole sample		
Element	wt%	wt% σ	Element	wt%	wt% σ	Element	wt%	wt% σ	Element	wt%	wt% σ
O	45.43	0.17	O	52.10	2.89	C	43.45	4.30	C	59.22	0.08
C	33.39	0.23	C	24.70	2.33	O	37.17	3.63	O	40.17	0.08
Si	10.85	0.04	Pb	7.26	1.17	Ti	5.30	0.59	S	0.30	0.00
Al	3.55	0.02	Ba	7.07	0.76	Si	4.62	0.49	Si	0.15	0.00
Ca	1.51	0.01	S	2.37	0.34	Ca	3.68	0.44	Cl	0.05	0.00
Fe	1.44	0.01	Cr	2.05	0.34	Br	3.12	0.54	Hg	0.05	0.01
Hg	0.99	0.03	Si	1.67	0.23	Fe	1.74	0.42	Fe	0.03	0.01
Mo	0.81	0.07	Ca	1.36	0.23	S	0.63	0.23	P	0.02	0.00
Ti	0.70	0.01	Al	0.78	0.22	Hg	0.30	0.73	Ti	0.01	0.00
Cl	0.55	0.01	Cl	0.41	0.28						
P	0.50	0.01	P	0.21	0.22						
S	0.27	0.02									
Total wt%	100		Total wt%	100		Total wt%	100		Total wt%	100	

Results are shown for a scan undertaken across the entirety of Sample 1 and for an individual Cr-bearing paint fleck and a Ti-bearing microplastic as highlighted in Fig. 2. The recorded wt% for each element, total wt%, and statistical error (wt% σ) are defined within the Oxford Instruments AZtec 6.1™ software. It should be noted that whilst elemental data for the plastic standards used in this study are shown here, this is strongly dependent on the individual standards chosen and their composition is not representative of the vast array of available polymer types.

In addition, the sample preparation and SEM-BEX method developed here allows for the characterisation of additional environmental material associated with the contaminant particles in one analytical run. Marine organisms such as gastropod molluscs, crustaceans, and diatoms are readily identified using the SEM-BEX imagery (Fig. 3). The physical characteristics of many of these organisms can be determined using the Fiji (ImageJ) workflow based on the elemental make-up of their skeletal component (e.g., calcium shells for gastropod molluscs vs. siliceous diatoms), with gastropod molluscs measuring ~ 30–50 µm^2^, diatoms measuring ~ 50–70 µm^2^, and crustaceans measuring ~ 1 mm^2^. Whilst an ecosystem assessment based on SEM-BEX imagery was beyond the scope of this study, the results presented here provide a basis for organism–contaminant relationship studies. For example, crustaceans or zooplankton are present in all samples aside from Sample 1, which contained the largest and most varied contaminant particles (Fig. 1) and was collected from surface waters of a river mouth near the harbour. These comparisons between large-scale observations taken during fieldwork (e.g., sampling location or environment) and microscopic observations obtained from SEM-BEX allow for data integration gaps to be bridged in determining whether the scarcity of an organism (e.g., zooplankton) is the result of particulate contamination and trace metal pollution in this location or simply that the brackish environment does not support these species.

## Discussion

SEM-BEX is an effective tool for detecting microplastics and other particles in samples taken from the marine environment without the time-consuming need for physical or chemical separation of individual contaminant particles from the associated suspended sediment and organic matter. Chemical separation steps can result in the removal of associated biofilms and cause other destructive effects (e.g., acid digestion)^17^. The system used in this study produces high-resolution (8343 × 7758 pixels) elemental maps up to 18× faster than standalone EDX detectors and useable X-ray output count rates of 900,000 cps (compared with the ~ 90,000 cps X-ray output achieved by the Oxford Instruments X-MaxN 150 mm EDX detector used in this study and others)^14^. This allows for high-sample throughput, with analysis times of ~ 20 min per sample using the parameters defined in this study. The streamlined preparation, analysis, and data interpretation methods presented here provide capability for rapid quantification of particles of potential concern for environmental studies and ecological risk assessment.

The combination of BSE imagery with elemental data from the SEM-BEX system is particularly useful for broad-scale environmental contaminant studies as it allows the user to accurately determine the physical characteristics of individual particles, including their size, shape, abundance, and surface topography. This is particularly useful for identifying and describing transparent or colourless microplastic films that are overwise easily overlooked in optical microscopy studies. Such information can be used in hydrodynamic models to track particle distribution from sources to sinks in aquatic environments (e.g., size and shape)^18^, budget calculations for contaminants in different environments (e.g., abundance)^19^, and inferences on the degradation of particles (such as microplastics) due to abrasion during transport or biological activity (e.g., surface topography and circularity index)^20^. Similarly, the individual element and all-element maps provided by SEM-BEX allow for the distinction of a broad-suite of manufactured contaminants (microplastics, paint flecks, rubber, etc.). This, in combination with optical microscopy, assists with distinct source-matching, as, for example, the abundant blue and yellow paint flecks and vulcanised rubber fragments identified in the samples analysed here, which are most likely locally sourced from cargo ships and fishing boats operating around Cát Bà Island that use these materials (e.g., blue and yellow painted hulls and masts; mooring tyres; Fig. 2). These applications again support the use of SEM-BEX as part of a wider toolkit for regional-scale environmental assessment that includes contextual field observations, optical microscopy, and particle-specific polymer-identification (e.g., Raman and fourier transform infrared spectroscopy [FTIR]). The high-resolution surface topography maps also allow for the identification and visual assessment of degraded particles prior to further FTIR or Raman spectroscopy. This is important because the degradation of particles during transport or exposure to the environment can lead to either the addition or removal of key markers used in the identification of specific polymer types (such as the addition of oxygen that may be used in the identification of PET).

Another strength of rapid SEM-BEX analysis is the ability to scan for and quantify toxic trace metals and other elements associated with contaminant particles, organic materials, and sediment present in each environmental sample (e.g., Hg, Cr, Ti; Fig. 2). The identification and impacts of such trace elements in individual particles has previously been shown using SEM–EDX, including studies on the adsorption of lead (Pb), cadmium (Cd), and zinc (Zn) to PET particles^21^, and the impact that flame-retardant additives (hexabromocyclododecane) have on copper (Cu), nickel (Ni), and Zn adsorption to microplastic particles in East Asia^22^. However, the proposed SEM-BEX workflow enables a more comprehensive environmental analysis through rapid scanning of both contaminant particles and other inorganic and organic material within the water column (such as suspended sediment or micro-organisms). The combined use of SEM-BEX with an attached standalone EDX detector (e.g., X-Max^N^ 150 mm Energy Dispersive X-ray detector) means that all trace metals present within a sample can be analysed in both a qualitative and quantitative fashion, whereby, SEM-BEX fast-scan maps provide a qualitative visual assessment of trace metal hotspots before direct analysis of those hotspots are performed using the attached EDX detector to obtain full quantification of trace metal abundance. For example, Sample 1 (Fig. 2) provides an indication that, whilst metals such as Cr and Ti are more concentrated in specific particles (e.g., Cl-bearing film and paint flecks; likely due to their introduction in manufacturing processes), Hg concentrations are more evenly distributed throughout the sample (Fig. 2), hence suggesting contamination throughout the water column that impacts all organic matter, contaminant particles, and sediment.

In addition, understanding the environmental contamination of marine and freshwater ecosystems can be enhanced using the novel SEM-BEX workflow as it provides integrated size, abundance, and elemental data for smaller contaminant particles (µm-scale), highlighting their potential impacts on organisms in single environmental samples. For example, the diatoms present in all samples analysed are comparable in size and shape to many of the smaller Cl-bearing microplastic fragments (~ 50–70 µm^2^, circularity index of 0.8–1; Fig. 3). Diatoms make up the diet of many of the zooplankton and molluscs present in the samples, such as krill, and microplastics are often incidentally ingested by these organisms^23^. The detection of these microplastic particles via SEM-BEX, therefore, helps to inform us on potential harm to the wider food web caused by the presence of microplastics and other contaminant particles (with humans ingesting ~ 10,000 microplastics from shellfish per year)^24^ alongside understanding the bioaccumulation of toxic trace metals in larger marine fauna that are shown to be adsorbed on microplastics (Fig. 2)^25^.

Despite these unique benefits of the Unity detector and SEM-BEX imaging, several limitations do exist. Most pronounced is the limited ability of SEM-BEX to determine individual microplastic polymer-types. Whilst we have shown the potential for identification of PVC, other chlorinated plastics, and additional contaminant particles (e.g., paint) using SEM-BEX, this is not as robust as previously utilised FTIR or Raman spectroscopy for polymer identification. Instead, this study proposes that SEM-BEX is most effective at determining the abundance of broad microplastics (regardless of polymer-type) and other particles within a sample along with assessing their physical and chemical characteristics (such as trace metal content). This fast-screening method allows for the identification of hotspots of microplastic abundance in the environment prior to further polymer identification studies (e.g., FTIR). In addition, whilst the fast-scanning SEM-BEX protocol presented here is efficient at mapping microplastics and other particles in the smaller end of the microplastic size range (e.g., ~ 5–50 µm; Fig. 2; Table 1), the resolution provided by this method (8343 × 7758 pixels) is not suitable for imaging nanoplastics (1 nm to 1 µm). Despite this, previous studies have shown that particles within the nanoplastic size range can be both physically and chemically characterised using SEM-BEX by focusing on a specific region and increasing both the scanning rates and resolution^26^.

**Fig. 4:**
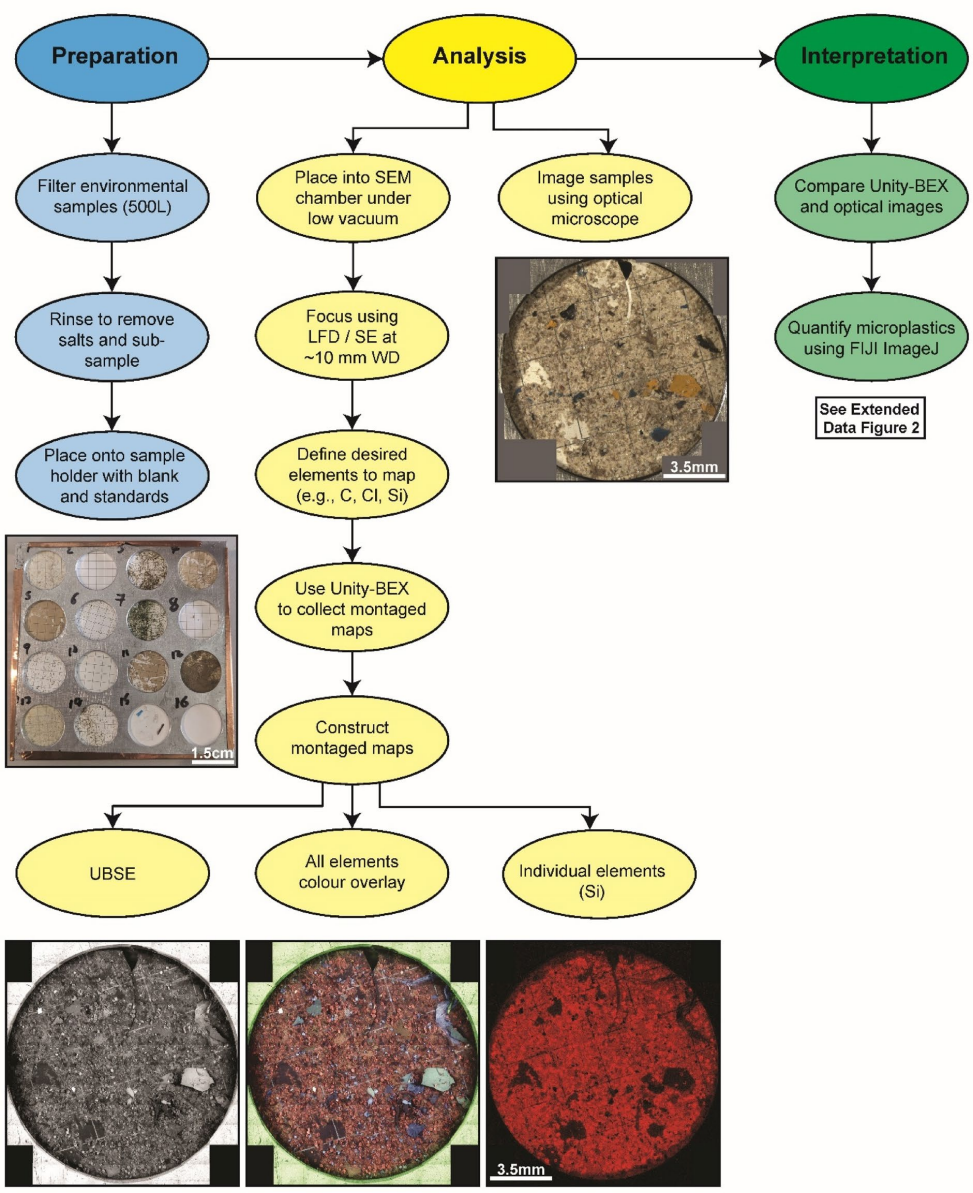
SEM-BEX analytical workflow. Proposed preparation, analysis, and initial interpretation workflow for the imaging of microplastics and other particles using a combined SEM-BEX and optical microscopy approach.

**Fig. 5:**
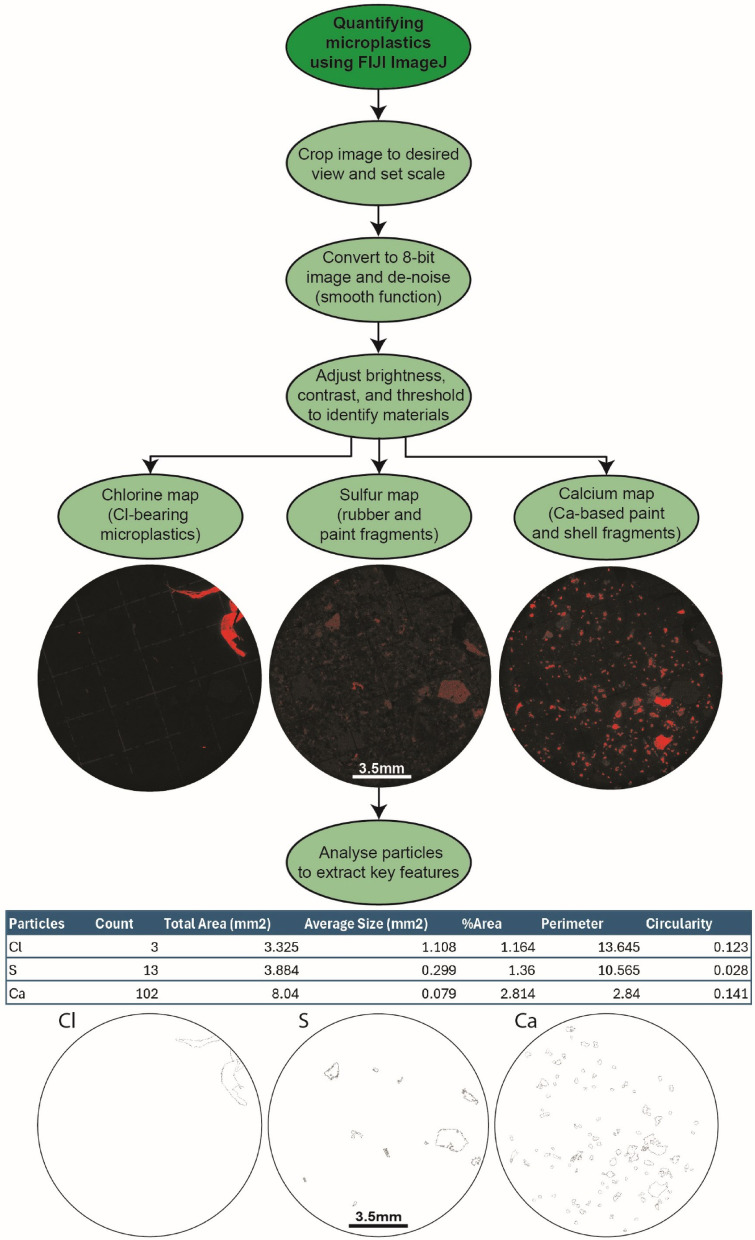
Semi-automated characterisation of particles. roposed semi-automated workflow for the characterisation of microplastics and other particles using Fiji (ImageJ) from SEM-BEX imagery.

Finally, our comparison between full automation and manual selection of particles (Table 1) demonstrates that over- and under-quantification during automated analyses of SEM images remains an issue, as identified by initial QEMSCAN studies^15^. Because of this, we recommend the use of a semi-automated approach in which, SEM-BEX imagery is supported by optical microscopy for further characterisation (e.g., true colour). This could be strengthened by integrating the workflow introduced in this study with image-based machine learning algorithms^27^ to identify contaminant particles of a distinct chemistry (e.g., automating the selection of PVC from Cl maps), where the model is trained by both manual particle selection and the semi-automated Fiji (ImageJ) workflow presented here (outlined in Figs. 4 and 5). Furthermore, the proposed workflow offers a rapid initial screening tool providing an overview of an entire environmental sample before more advanced and labour intense particle-specific analysis ensues. The proposed screening step will include immediate quantification of elemental abundances using SEM-BEX and standalone EDX detectors within the same SEM before the sample is extracted for further Raman or FTIR spectroscopy to measure the distribution of specific polymer types (e.g., PS, PP, PE)^28^.

## Conclusions

SEM-BEX is an efficient and effective technique for understanding the presence of contaminant particles (including some microplastics) in the environment at scale, allowing for their quantification. The sample collection and imaging protocol described here preserves organic matter and sediment from within the water column, allowing the interaction of contaminant particles and micro-organisms to be further studied, and trace metal distribution amongst organic, non-organic, and contaminant particles to be assessed. The increased analytical speeds and imaging resolution of SEM-BEX compared to standalone SEM–EDX makes it an appropriate tool in the broad-scale environmental impact assessment of large regions (such as Viet Nam). SEM-BEX allows initial observations on trace element distribution and contaminant abundance to be determined for large sample sets prior to conducting detailed analysis via vibrational spectroscopy (e.g., Raman) to determine microplastic polymer-types or further trace metal quantification using mass-spectrometry.

## Methods

### SEM-BEX imaging

SEM imaging was undertaken at the Centre for Environmental Scanning Electron Microscopy (CESEM) at Heriot-Watt University Edinburgh, UK. The Unity detector from Oxford Instruments was used for combined backscattered electron and EDX imaging (BEX) attached to an FEI Quanta 650 FEG Scanning Electron Microscope (SEM). Alongside this, an Oxford Instruments X-Max^N^ 150 mm Energy Dispersive X-ray (EDX) detector was utilised for additional quantitative EDX analysis of regions of interest where necessary. Environmental samples from the Cát Bà Islands, Viet Nam were collected from the sea surface (~ 0 mbsl), middle water depth (~ 2–5 mbsl), and bottom waters (~ 5–10 mbsl) in March and July of 2023 and filtered from 600 L of water on to a 80 µm pore size metal filter, before transferring any particulate matter to Mixed Cellulose Esters (MSE) membrane filters with pore sizes of 0.22 to 0.45 µm by filtering 25 mL of concentrate and further rinsing with sterile 0.2 M phosphate-buffered saline (PBS), before freezing at − 80 °C during storage. Field blanks were also prepared in triplicate for each sample site to assess for contamination during sample collection, these followed the same procedure as environmental samples whereby 25 mL of MilliQ was substituted for environmental water and used during the rinsing of a fresh metal filter on to a fresh MSE membrane filter. During sample preparation all samples are further rinsed with MilliQ to remove any salts from the sample matrix, air-dried, and cut into 15 mm rounds before being mounted uncoated onto a custom-built aluminium mount with 16 sample slots (Fig. 4). Fourteen slots were used for unknown samples, one slot was occupied by standard materials of known composition, and another by a blank filter paper that is kept uncovered in the preparation area to collect any environmental contaminants that might be introduced during sample preparation. Careful note was taken of the colour and fibre types of clothes worn during sample preparation, along with any other potential sources of contamination.

For automated SEM-BEX mapping, the SEM is operated in low-vacuum mode with a chamber pressure of 0.82 torr. A working distance of ~ 10–11 mm is used along with a spot size of 5, a magnification of 100×, a dwell time of 10 µs, an aperture strip position of 1, and an operating voltage of 20 kV. The large field detector (LFD) was used for initial focussing and brightness/contrast adjustment of the sample image. To allow automated mapping and high sample throughput the custom cartography feature in the Oxford Instruments AZtec 6.1™ software was used to define individual sample areas and select pre-defined elements relevant to this study (carbon [C], chlorine [Cl], sulfur [S], phosphorous [P], silica [Si], aluminium [Al], calcium [Ca]). In addition, elemental maps and concentrations of cadmium [Cd], lead [Pb], titanium [Ti], mercury [Hg], zinc [Zi], cobalt [Co], copper [Cu], and nickel [Ni] were further quantified during post-processing to assess any trace metals present.

### Standard material characterisation

During analysis a fabricated sample (positive control) containing known plastic types was imaged alongside every 14 unknown samples (based on the custom-built sample holder used in this study that allows analysis for 16 individual samples—Including 14 unknowns, 1 positive control, and 1 negative control). This fabricated sample contained both virgin plastics of known types with defined elemental abundances (e.g., atomic % of carbon, oxygen, etc.) obtained from Goodfellow, Thermo Fisher, and Araldite, alongside used plastics of known composition to better mimic plastic types and colours that may be encountered in the unknown environmental samples. Of these, the virgin plastics include a clear Polyurethane (PU) granule, white Polyethylene Terephthalate (PET) powder, a white Polystyrene (PS) ball, a brown High-Density Polyethylene (HDPE) fragment, and a clear epoxy resin fragment. Additional environmental mimicking plastics include blue Polypropylene (PP) fibres, white polyester-cotton blend fibres, a clear Polyvinyl Acetate (PVA) fragment, a clear Polyethylene (PE) fragment, white polyester fibres, a clear Polyvinyl Chloride (PVC) fragment, and white Nylon that serves as the sample base and mounting material (Fig. 1). These fabricated standards allow for immediate qualitative correlation between standard plastics and unknown plastics across a sample based on colour intensity in both BSE (greyscale) and EDX (element-defined colours), using the SEM-BEX fast scanning function. This can be combined with full quantification of elemental abundance if the SEM-BEX is deployed in unison with a standalone EDX detector (such as the Oxford Instruments X-MaxN 150 mm Energy Dispersive X-ray detector used in this study), this is most effective in the identification of the PVC and PET standards due to the presence of Cl and elevated O, respectively. However, it should be noted that these standard materials only represent an initial test case for the SEM-BEX method and are not necessarily representative of the vast array of polymer-types and colourations that may be present in the natural environment.

### Optical microscopy

To support the complete recognition of all particles in each sample optical microscope images were taken using a Leica S8APO (Fig. 4). These images are used as a secondary confirmation tool to assess the accuracy of BEX in identifying specific particles and can also be helping in distinguishing particles of different colour (e.g., blue vs yellow fragments; Fig. 4). In addition, the fast-scanning SEM-BEX technique and Fiji (ImageJ) characterisation applied here has limitations in distinguishing different fibre types (e.g., cotton, polyester, fibrous organic matter), as these materials appear as indistinguishable lines of carbon at the resolution achieved on the elemental maps (Fig. 1). Distinction between fibre types can be achieved within the fast-scanning framework by assigning true colours and morphologies via comparison with optical microscopy (as outlined above) or by imaging individual fibres at higher magnifications and characterising their microstructure on a case-by-case basis using SEM-BEX.

### Image manipulation

Following SEM-BEX fast scanning and collection of all BSE and pre-defined EDX data, post-processing and data manipulation took place using the Oxford Instruments AZtec 6.1™ software. This allows montaging of all data into combined element maps (Figs. 1 and 4), selection of additional elemental data to be shown where necessary (e.g., Cd, Pb, Ti, Hg, Zi, Co, Cu, and Ni to determine trace metal adsorption by plastics), and further elemental quantification of particles using an attached Oxford Instruments X-MaxN 150 mm Energy Dispersive X-ray (EDX) detector. Semi-automated characterisation took place using the Fiji (ImageJ) software^16^ that further allowed quantification of particle abundance, size, and shape. The method for semi-automated extraction of particle data is show in Fig. 5 and involves an iterative workflow of adjusting the brightness and contrast for different elemental scans before applying a threshold that Fiji (ImageJ) uses to automatically draw around the particle (using the ‘analyse particles’ function) thereby producing predefined physical characteristics (in this case: count, total area, average size, %area, perimeter, and circularity) for each individual particle. These data have several uses, for example, circularity data can then be converted into a qualitative shape scale to assess levels of particle breakdown in the environment (e.g., rough vs. smooth and fragmented vs. whole). This process is most efficient when defining the physical characteristics of element-specific materials such as PVC (Chlorine), vulcanised rubber (Sulfur), or marine shells (Calcium), here the individual element maps provide confidence in the selection of threshold values prior to Fiji (ImageJ) automated analysis as other materials are already excluded. However, quantifying the entire particle load of a sample using Fiji (ImageJ) and SEM-BEX imagery does require use of optical imagery to cross-check particle location.

## Data Availability

Additional datasets generated during and/or analysed during the current study are available from the corresponding author on reasonable request. They will also be available for download from the British Oceanographic Data Centre (BODC) following a 2-year embargo period (from March 2027-onwards).
